# The role of third-line chemotherapy in recurrent or metastatic gastric cancer: A cohort study with propensity score matching analysis

**DOI:** 10.1097/MD.0000000000012588

**Published:** 2018-09-28

**Authors:** Yong Won Choi, Mi Sun Ahn, Geum Sook Jeong, Hyun Woo Lee, Seong Hyun Jeong, Seok Yun Kang, Joon Seong Park, Jin-Hyuk Choi, Seung Soo Sheen

**Affiliations:** a Department of Hematology-Oncology; b Department of Pulmonary and Critical Care Medicine, Ajou University School of Medicine, Suwon, Korea.

**Keywords:** gastric cancer, overall survival, recurrent or metastatic, third-line chemotherapy

## Abstract

In recurrent or metastatic gastric cancer, second-line chemotherapy is generally recommended in current guidelines. Although third-line therapy is often performed in daily practice in some countries, there are only a few reports about its benefits.

A retrospective review was conducted on 682 patients who underwent at least first-line chemotherapy for recurrent (n = 297) or primary metastatic (n = 385) disease. Clinicopathological characteristics and overall survival (OS) were analyzed according to lines of chemotherapy.

One hundred sixty-seven patients (24.5%) underwent third- or further-line therapy. Third- or further-line therapy was frequently performed in patients with young age (<70) (*P* < .0001), Eastern Cooperative Oncology Group (ECOG) performance status (PS) 0 or 1 (*P* < .0001), surgical resection before first-line therapy (*P* = .007), and first-line combination regimen (*P* = .001). The median OS for all patients after the initiation of first-line therapy was 10 months. The median OS of patients who received third- or further-line therapy was significantly longer than that of patients who received second- or lesser-line therapy (18 vs 8 months, *P* < .0001). The multivariate analysis revealed that third- or further-line therapy was independently associated with favorable OS (hazard ratio = 0.58, *P* < .0001). Moreover, patients who received third- or further-line therapy demonstrated better OS both in univariate (*P* = .002) and multivariate (*P* < .0001) analysis even after propensity score matching using baseline characteristics. The median OS after the start of third-line chemotherapy was 6 months. In addition, ECOG PS 0 or 1 at the initiation of third-line therapy (*P* < .0001) and surgical resection (*P* = .009) were independently associated with longer OS after third-line therapy.

The current study suggests that third-line therapy could be recommended for recurrent or metastatic gastric cancer patients with good PS after progression from second-line chemotherapy in clinical practice.

## 1 Introduction

Gastric cancer is the second most common malignancy in Korea and the third leading cause of cancer-related death worldwide including Korea.^[1,2]^ For patients with recurrent or metastatic stage IV gastric cancer, palliative chemotherapy is the standard of care. However, almost all patients eventually experience disease progression during or after completion of first-line chemotherapy. After the failure of first-line therapy, second-line chemotherapy is recommend for patients with a good performance status (PS) based on the results of the phase III trials demonstrating overall survival (OS) benefit.^[3–6]^ Moreover, third- or subsequent-line therapy in selected patients is commonly performed in clinical practice in some countries, including Korea, despite the lack of randomized trials supporting the benefit of third-line chemotherapy using cytotoxic agents.^[7,8]^

Consequently, determining whether third-line therapy is more beneficial than best supportive care and identifying patients who will benefit from third-line therapy have become important clinical issues. However, to our knowledge, even retrospective study has not been reported comparing the outcome of patients with gastric cancer who underwent third- or further-line therapy with that of those who received second- or lesser-line therapy in terms of OS from the initiation of first-line chemotherapy using the cohort that included all the patients who received palliative chemotherapy during the defined period. Therefore, we retrospectively compared the outcomes between recurrent or primary metastatic gastric cancer patients who received third- or further-line therapy and those who received second- or lesser-line therapy as well as analyzed the clinicopathological characteristics affecting prognosis.

## 2 Patients and methods

### 2.1 Study population

We retrospectively identified all patients with recurrent or metastatic gastric cancer who had started first-line palliative chemotherapy between January 2004 and December 2014 at our institution. The criterion for eligibility was histologically documented recurrent or primary metastatic gastric cancer. In patients with primary metastatic disease, American Joint Committee on Cancer stage IV^[9]^ patients with distant metastasis or patients with gross residual disease after surgical resection were included. Among stage IV diseases, patients with distant abdominal lymph node metastasis (e.g., retropancreatic or mesenteric) were excluded if complete resection of gross tumor was performed. If adequate chemotherapy information was available, patients who had started first-line chemotherapy at other hospitals during this period and received further therapy at our institution were included. Patients who were transferred to other hospitals for chemotherapy during or after first- or second-line therapy at our institution were excluded if further treatment information was unavailable. This research protocol was approved by the institutional review board (IRB) of Ajou University Hospital, Suwon, Korea (IRB approval no. AJIRB-MED-MDB-16-022).

### 2.2 Clinical review

A retrospective review of the clinical information of eligible patients was performed. Data on the gastric cancer patients, including patient characteristics [sex, age, PS based on the Eastern Cooperative Oncology Group (ECOG) performance scale, histology, disease status at diagnosis, peritoneal and liver metastasis, palliative surgical resection before initiation of first-line chemotherapy, chemotherapy regimens, and total chemotherapy lines] and survival information, were collected. For histologic subclassification, pathologic information on primary tumor of stomach was used in both primary metastatic and recurrent disease. In patients with local recurrence, histology was classified according to the pathology report on the recurrent stomach lesion if available.

### 2.3 Statistical analysis

OS was calculated using the Kaplan-Meier method. OS was defined as the time from the starting day of the first- or third-line chemotherapy to death. Data on the survivors were censored at the last follow-up. The differences between the survival curves were analyzed by the log-rank test. Fisher exact test was used to compare the different groups for categorical variables. The Cox proportional hazards regression model was used to determine the joint effects of several variables on survival. Factors with *P* values <.1 in the univariate analysis were included in the Cox proportional hazards regression model. All statistical analyses were performed 2 sided with SPSS version 23.0 for Windows (IBM Corp., Armonk, NY).

Propensity score matching (PSM) was used to minimize the selection bias by balancing covariates that may be associated with the outcome. In the present study, the 1:1 nearest neighbor matching was performed using SPSS version 23.0 for Windows.

## 3 Results

### 3.1 Patient characteristics

Among the 692 patients who started first-line palliative chemotherapy for recurrent or primary metastatic gastric cancer at our institution, 23 patients who were transferred to other hospitals for chemotherapy without follow-up treatment information were excluded and 13 patients who had started first- or further-line therapy at other hospitals were included, thus leaving 682 patients for analysis. Most patients underwent first-line chemotherapy as routine clinical practice. However, 14 (2.1%) patients received first-line therapy in clinical trials (12 patients: a phase III trial comparing 3-weekly and 5-weekly cisplatin plus S1 chemotherapy; 2 patients: phase II or III trials evaluating the efficacy of adding targeted agents to chemotherapy in human epidermal growth factor receptor 2–positive advanced gastric cancer).

Table 1 summarizes the patients’ clinicopathological characteristics. Among the 682 patients, 475 (69.6%) were men, 124 (18.2%) were 70 years or older, 604 (88.6%) were in ECOG PS 0 or 1, and 188 (27.6%) had poorly differentiated adenocarcinoma as the most prevalent histological type. Among the 297 (43.5%) patients with recurrent disease, 267 had received adjuvant chemotherapy. A total of 314 (46.0%) and 156 (22.9%) patients had peritoneal and liver metastasis, respectively, and 35 (5.1%) patients had both liver and peritoneal metastasis. Palliative surgical resection (gastrectomy: 81; metastasectomy: 42; both: 14) before first-line therapy was performed in 137 (20.1%) patients. First-line chemotherapy was combination for 521 (76.4%) patients [5-FU/leucovorin/oxaliplatin (FOLFOX): 350; S1/cisplatin (SP): 74; XELOX (capecitabine/oxaliplatin): 23; capecitabine (or 5-FU)/cisplatin/trastuzumab: 10; 5-FU/leucovorin/irinotecan (FOLFIRI): 10; others: 54] and single agent for 161 (23.6%) patients (S1: 142; capecitabine: 6; UFT: 5; others: 8). Overall, 194 (28.4%) and 167 (24.5%) of all patients received second- and third- or further-line therapy, respectively. Third-line chemotherapy was combination for 85 (50.9%) patients (FOLFIRI: 53; FOLFOX: 13; SP: 7; paclitaxel/cisplatin: 4; irinotecan/cisplatin: 4; others: 4) and single agent for 82 (49.1%) patients (docetaxel: 28; S1: 27; UFT: 15; paclitaxel: 7; others: 5). Among the 167 patients with third- or further-line therapy, 58 (34.7%) received fourth- or further-line chemotherapy (maximum: seventh line).

**Table 1:**
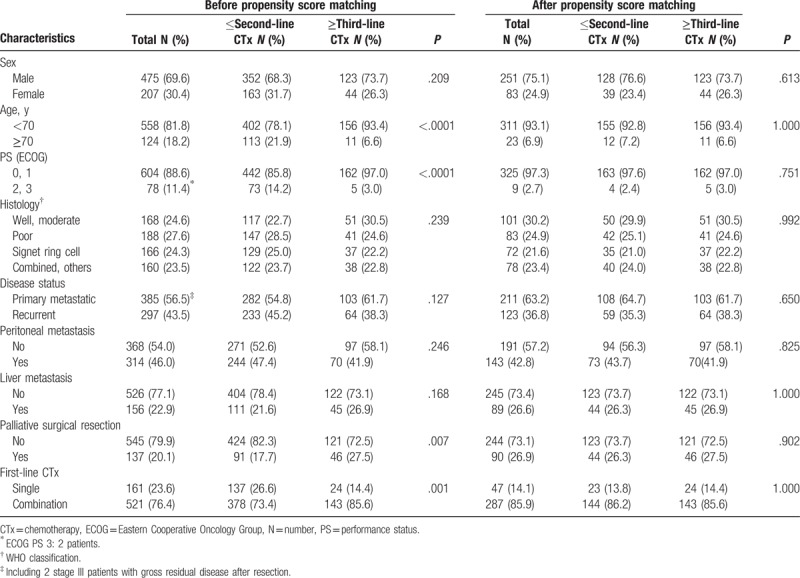
Patient characteristics at the initiation of first-line chemotherapy.

In the baseline characteristics, patients who received third- or further-line therapy were associated with a high proportion of young age (<70) (*P* < .0001), good PS (*P* < .0001), palliative surgical resection before first-line therapy (*P* = .007), and first-line combination regimen (*P* = .001) in comparison with those who underwent second- or lesser-line chemotherapy (Table 1). To control the selection bias, the patient characteristics at the initiation of first-line chemotherapy were used as covariates for PSM. The covariates were well balanced after 1:1 PSM without statistically significant difference between patients who received third- or further-line therapy and those who underwent second- or lesser-line chemotherapy (Table 1).

### 3.2 Overall survival

**Figure 1:**
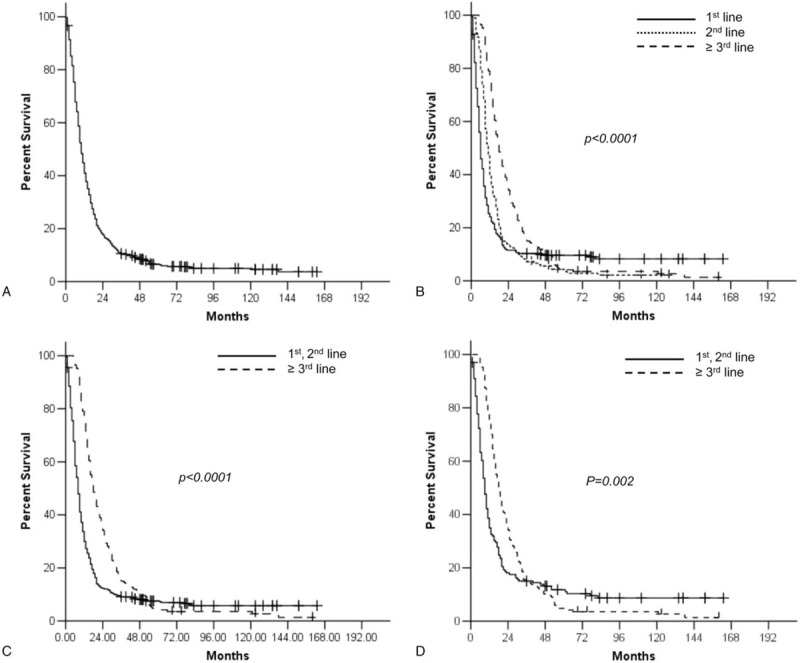
Overall survival from the start of first-line chemotherapy for all patients (A), and according to the number of chemotherapy lines before (B), (C) and after propensity score matching (D).

The median follow-up duration was 77 months (36–163 months) for the survivors. Only 1 patient was lost to follow-up for survival status at 1 month after the initiation of first-line chemotherapy. This patient's survival data were censored at the last follow-up time. Thirty-nine (5.7%) patients were still alive at the time of the last follow-up. The median OS for all patients after the initiation of first-line therapy was 10 months (Fig. 1A). The Kaplan-Meier survival curve for OS from the start of first-line therapy according to the chemotherapy lines, as presented in Figure 1B, showed a statistically significant difference. The median OS of patients who received third- or further-line therapy was significantly longer than that of patients who received second- or lesser-line therapy (18 vs 8 months, *P* < .0001, Fig. 1C). In addition, patients who underwent palliative surgical resection before first-line therapy (*P* < .0001) and first-line combination chemotherapy (*P* < .0001) showed longer median OS. Old age (70 or more) (*P* = .024), ECOG PS 2 or more (*P* < .0001), signet ring cell histology (*P* = .040), and presence of peritoneal metastasis (*P* = .001) were associated with poor OS in the univariate analysis (Table 2).

**Table 2:**
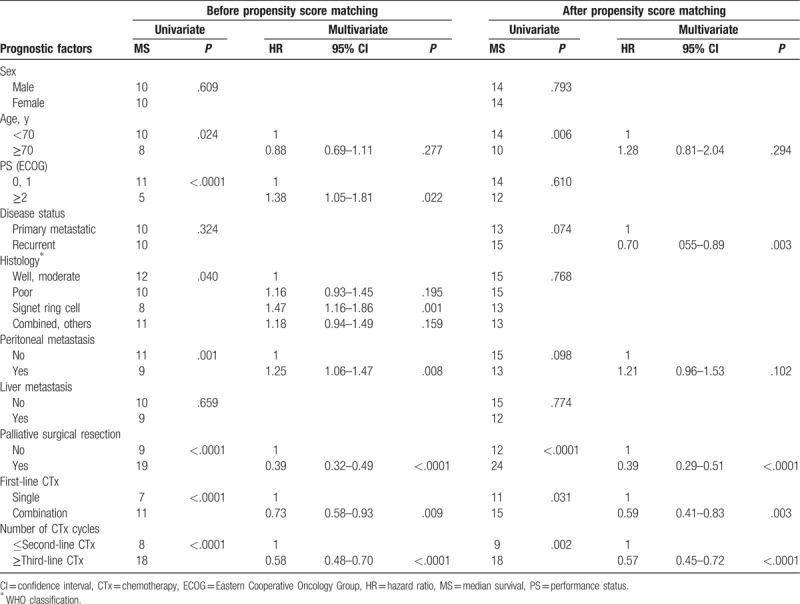
Univariate and multivariate analysis of overall survival for all patients from the start of first-line chemotherapy.

The multivariate analysis revealed that third- or further-line therapy was independently associated with favorable OS (hazard ratio = 0.58, *P* < .0001), along with surgical resection (*P* < .0001) and first-line combination regimen (*P* = .009), whereas ECOG PS 2 or more (*P* = .022), signet ring cell histology (*P* = .001), and peritoneal metastasis (*P* = .008) were independent prognostic factors of poor OS (Table 2). Even after PSM, third- or further-line therapy was associated with better OS (18 vs 9 months, *P* = .002), compared with second- or lesser-line therapy in univariate analysis (Fig. 1D), with independent favorable prognostic significance (hazard ratio = 0.57, *P* < .0001) in multivariate analysis (Table 2).

### 3.3 Overall survival after third- or further-line therapy

**Figure 2:**
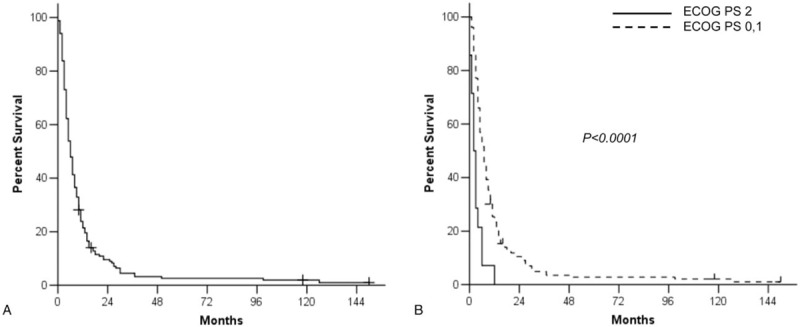
Overall survival from the initiation of third-line therapy for all patients (A) and according to performance status (PS) at the start of third-line therapy (B).

The median OS after the initiation of third-line therapy was 6 months (Fig. 2A). In the univariate analysis, patients with good PS (ECOG PS 0 or 1) at the initiation of third-line therapy demonstrated longer OS (7 vs 2 months, *P* < .0001, Fig. 2B). In addition, younger age (<70) (*P* = .040) and palliative surgical resection before first-line therapy (*P* = .019) were associated with longer OS after third-line therapy. Other clinicopathologic characteristics including third-line chemotherapy regimens (single vs combination) were not associated with the OS of patients (Table 3). Furthermore, good PS at the initiation of third-line therapy (*P* < .0001) and palliative surgical resection (*P* = .009) were independent favorable prognostic factors in multivariate analysis (Table 3).

**Table 3:**
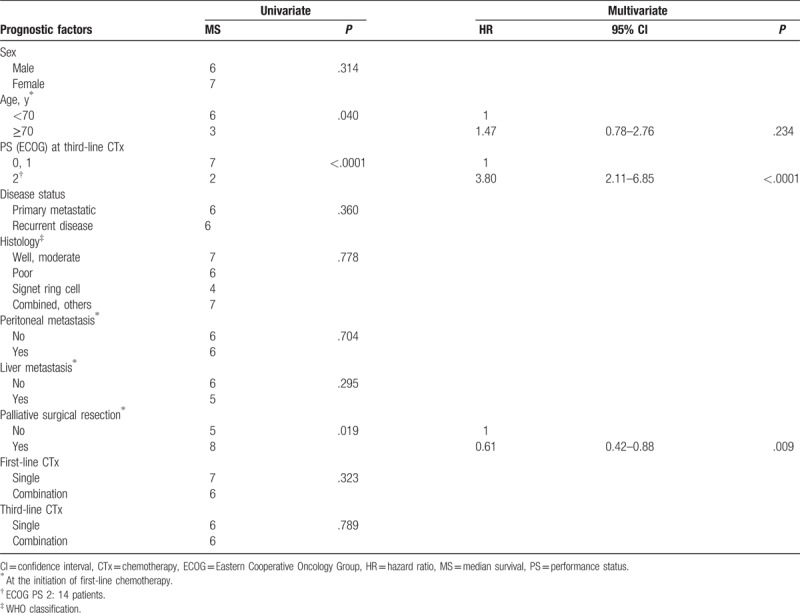
Univariate and multivariate analysis of overall survival from the start of third-line chemotherapy.

In addition to OS, to determine the change of PS after third-line chemotherapy, ECOG PS of patients around 1 month (20–40 days) after the start of chemotherapy was analyzed. Among 153 patients with PS 0 or 1 at the time of initiation of third-line chemotherapy, 128 (83.7%) patients maintained the initial good PS, whereas 11 (7.2%), 5 (3.3%), 7 (4.6%), and 2 (1.3%) were in PS 2, 3, 4, and 5 (death), respectively. On the contrary, in cases of 14 patients with PS 2, PS was 1 in 2 (14.3%) patients, 2 in 4 (28.6%), 3 in 3 (21.4%), 4 in 1 (7.1%), and 5 in 4 (28.6%), after third-line chemotherapy.

## 4 Discussion

The present study analyzed patients with recurrent or primary metastatic gastric cancer who had started first-line chemotherapy since 2004 at our institution, as third-generation agents such as oxaliplatin and S1^[10–12]^ have become reimbursable for palliative chemotherapy from the Korean national health insurance system beginning 2004. In this study, 194 (28.4%) and 167 (24.5%) patients were treated with second- and third- or further-line therapy, respectively. The proportion of patients treated with third- or further-line therapy was comparable with those in previous reports from other Korean institutions.^[7,8]^ This relatively high frequency of third-line chemotherapy despite the lack of evidence suggesting third-line therapy as the standard care can be explained by the practice pattern in Korea. Most clinicians and patients with gastric cancer in Korea tend to continue chemotherapy in third- or further-line setting. In addition, the reimbursement from the Korean national health insurance system can be helpful in receiving third- or further-line therapy.

In this study, median OS (10 months) of all patients after the initiation of first-line chemotherapy was comparable with those in previous studies with a large number of patients including Korean institutions.^[7,10–15]^ The median OS of patients who received third- or further-line therapy was significantly longer than that of patients who received second- or lesser-line therapy. Moreover, third- or further-line therapy itself was independently associated with a favorable outcome in the multivariate analysis. The median OS of 18 months in patients who underwent third- or further-line therapy seemed to be quite encouraging. Nonetheless, the proportion of patients with younger age, good PS, palliative surgical resection, and combination regimen at the initiation of first-line chemotherapy was relatively high in the third- or further-line therapy group. These findings suggest the possibility that patients in relatively healthy status or with low tumor burden at the initiation of first-line chemotherapy have more chance of being selected for third-line chemotherapy. Therefore, to overcome bias, PSM analysis was performed by using patient characteristics before the start of first-line chemotherapy as covariates. The survival benefit of third- or further-line therapy was consistently demonstrated in univariate and multivariate analysis even after PSM. However, only randomized trials could definitely answer the question of whether this favorable outcome was due to the effect of third-line chemotherapy itself or the patients’ characteristics associated with favorable outcomes.

The median survival was 6 months in recurrent or metastatic gastric cancer patients from the start of third-line chemotherapy. Among various clinicopathologic characteristics, good PS at the initiation of third-line therapy was significantly associated with favorable OS both in univariate and multivariate analysis. Patients with ECOG PS 2 demonstrated extremely poor median OS of 2 months. Moreover, approximately 60% of patients with ECOG PS 2 at the initiation of third-line chemotherapy experienced deterioration of PS or early death, whereas 84% of PS 0 or 1 patients maintained the initial good PS, around 1 month after start of chemotherapy. These findings suggest that patients with poor PS should not be considered for third-line therapy with cytotoxic agents. In addition, patients with palliative surgical resection before first-line chemotherapy also showed significantly favorable OS after third-line therapy. This result suggests that third-line chemotherapy could be more beneficial for patients with a lower tumor burden.

The median OS of third-line chemotherapy from other studies was 3.6 to 7.5 months.^[7,8,16–22]^ Most published studies were retrospective analyses or small phase II trials from Korea or Japan and usually used taxanes and irinotecan as single or combination regimens.^[7,8,16–22]^ In 2 phase II trials with docetaxel from Korea and 1 phase II trial with paclitaxel from Japan, the median OS was 3.6, 4.7, and 6.7 months, respectively,^[16,17,22]^ and 2 retrospective studies using irinotecan from Japan showed a median OS of 4 and 6 months, respectively.^[19,21]^ In a relatively large retrospective study including 158 patients from Korea, FOLFIRI regimen showed a median OS of 5.6 months.^[18]^ In the only western study from Italy, the median OS of FOLFIRI regimen as a third-line therapy was 7.5 months.^[20]^ In the present study, there was no difference in OS from the initiation of third-line therapy between single and combination regimens. Therefore, single-agent regimen can be recommended in third-line therapy given the relatively high toxicity of combination chemotherapy.

To our knowledge, the phase III trial evaluating the efficacy of cytotoxic agents has never been performed in a third-line palliative chemotherapy setting. However, in recently reported randomized phase III trials, apatinib, a vascular endothelial growth factor receptor 2 tyrosine kinase inhibitor,^[23]^ and nivolumab, an antiprogrammed death 1 monoclonal antibody,^[24]^ acting as an immune checkpoint inhibitor, as third- or further-line therapy demonstrated OS benefit compared with placebo. In addition, in a phase II trial, pembrolizumab, another antiprogrammed death 1 monoclonal antibody, showed favorable clinical activity including 5.6 months of median OS in advanced gastric cancer patients who experienced disease progression after 2 or more lines of therapy.^[25]^ Based on these phase II and III trials about immune check point inhibitors in gastric cancer, both nivolumab and pembrolizumab have been becoming available in clinical practice as third- or further-line therapy after approval in some countries including Korea. It is clinically meaningful that the median OS of the apatinib, nivolumab, and pembrolizumab groups from these 2 trials (6.5, 5.3, and 5.6 months, respectively) is comparable with that of the third- or further-line chemotherapy group in our study.^[23–25]^

In terms of application of third-line therapy in daily practice for recurrent or metastatic gastric cancer patients, either immune check point inhibitors or cytotoxic chemotherapy should be considered in patients who experienced progression after second-line therapy while maintaining good PS according to the approval and reimbursement status of agents in each country. The median OS of 6 months after third-line chemotherapy with cytotoxic agents in the current study cohort is almost comparable to that (5.8 months) of patients who received pembrolizumab as third-line therapy in the phase II trial including only ECOG PS 0 or 1 patients. This finding suggests that chemotherapy with cytotoxic agents is also a valid option as third-line therapy.

To our knowledge, the present study is the first report to compare the outcomes of patients treated with third- or further-line palliative chemotherapy with those of patients who underwent second- or lesser-line chemotherapy, analyzing OS from the start of first-line therapy. Almost all previous retrospective analyses or clinical trials evaluated the outcome of patients from the initiation of third-line chemotherapy.^[7,8,16–22]^ In addition, the analysis of all patients who underwent palliative chemotherapy in a single institution during the defined period with mature follow-up (minimum follow-up duration of survivors: 36 months) in the present study could reflect the treatment outcomes of real-world clinical practice. Furthermore, the present study also demonstrated potential benefit of third-line chemotherapy using cohort with minimal selection bias after PSM.

However, the present study has several limitations. First, this work is a retrospective analysis from a single institution. Second, variable chemotherapy regimens were used in several therapy lines. Third, no patient received immune check point inhibitor as third-line therapy due to the time frame of the study. Fourth, as trastuzumab has been available in routine practice since June 2011 in Korea, the number of patients treated with first-line chemotherapy including trastuzumab was small in the entire study population. Finally, to minimize the selection bias, it is ideal to compare the outcome between third- or further-line therapy and second- or less-line therapy groups using PSM of patient characteristics at the time of progression after second-line therapy. However, such analysis was almost impossible due to the retrospective nature of the study.

In conclusion, nearly one fourth of the recurrent or metastatic gastric cancer patients who had undergone palliative chemotherapy received third-line therapy with an encouraging median OS of 18 months after the initiation of first-line therapy in the present study. Since the number of available cytotoxic and molecular targeted agents is increasing, more patients will become candidates for third- or further-line therapy. Therefore, further prospective randomized trials are essential to more clearly define the role of third-line chemotherapy including optimal regimen for patients with recurrent or metastatic gastric cancer. Nonetheless, the current study suggests that third-line therapy could be recommended for recurrent or metastatic gastric cancer patients with good PS after progression from second-line chemotherapy in clinical practice.

## Author contributions

**Conceptualization:** Seok Yun Kang, Jin-Hyuk Choi.

**Data curation:** Yong Won Choi, Mi Sun Ahn, Geum Sook Jeong, Hyun Woo Lee, Seong Hyun Jeong, Seok Yun Kang, Joon Seong Park, Jin-Hyuk Choi, Seung Soo Sheen.

**Formal analysis:** Yong Won Choi, Jin-Hyuk Choi.

**Funding acquisition:** Jin-Hyuk Choi.

**Investigation:** Jin-Hyuk Choi.

**Supervision:** Seok Yun Kang, Jin-Hyuk Choi, Seung Soo Sheen.

**Writing – original draft:** Yong Won Choi, Jin-Hyuk Choi.

**Writing – review and editing:** Yong Won Choi, Seok Yun Kang, Jin-Hyuk Choi.
